# Supplementary material for: Intramolecular crossover from unconventional diamagnetism to paramagnetism of palladium ions probed by soft X-ray magnetic circular dichroism
Source: Commun Chem. 2020 Jul 31;3:96. doi: 10.1038/s42004-020-0327-9 (PMC9814631; doi:10.1038/s42004-020-0327-9)
Supplement: Supplementary file 1 — Supplementary Information [file 42004_2020_327_MOESM1_ESM.pdf]

Supplementary Information

Intramolecular crossover from unconventional diamagnetism to paramagnetism of palladium ions probed by soft X-ray magnetic circular dichroism

Alevtina Smekhova,[1,#] Detlef Schmitz,[2] Natalya V. Izarova,[1] Maria Stuckart,[1,3,†] S. Fatemeh Shams, [1] Konrad Siemensmeyer,[2] Frank M. F. de Groot,[4] Paul Kögerler, [1,3] Carolin Schmitz-Antoniak[1]*

[1]Peter-Grünberg-Institut (PGI-6), Forschungszentrum Jülich, 52425 Jülich, Germany

[2]Helmholtz-Zentrum Berlin für Materialien und Energie, Albert-Einstein-Str. 15, 12489 Berlin, Germany

[3]Institut für Anorganische Chemie, RWTH Aachen University, Landoltweg 1, 52074 Aachen, Germany

[4]Inorganic Chemistry and Catalysis Group, Debye Institute for Nanomaterials Science, Utrecht University, Universiteitsweg 99, Utrecht 3584 CG, The Netherlands

[#] present address: Helmholtz-Zentrum Berlin für Materialien und Energie, Albert-Einstein-Str. 15, 12489 Berlin, Germany

[†]present address: Department Chemie- und Bioingenieurwesen, Lehrstuhl für Chemische Reaktionstechnik (CRT), Friedrich-Alexander Universität Erlangen-Nürnberg, Egerlandstr. 3, 91058 Erlangen, Germany

* c.schmitz-antoniak@fz-juelich.de

Supplementary Note 1: XANES and XMCD of oxygen

The fine structure of the XANES at the oxygen K edge is exemplarily shown for oxygen in cobalt ferrite ($CoFe_2O_4$) in **Supplementary Figure 2**. The features A' and B' correspond to oxygen p states hybridised with the 3d states of Co and Fe in octahedral and tetrahedral symmetry [26]. In this energy range, the oxygen XMCD exhibits a negative and a positive peak corresponding to the parallel or antiparallel alignment of the magnetic moments of 3d metal ions to the external magnetic field on octahedral and tetrahedral lattice sites, respectively.

Contrary, in the case of the polyoxopalladates only one polarisation of oxygen is expected leading to one XMCD peak.

Peaks C' and D' correspond to oxygen hybridization with s,p states of the metal ions, while E' and F' are related to scattering [27,28].

Supplementary Note 2: CTM4XAS simulations - Parameters and different symmetries

All parameters that have been used for the CTM4XAS simulations presented in the manuscript are summarized in **Supplementary Table 1**. The crystal field parameters 10Dq, Ds, and Dt describe the symmetry of the environment around the Pd ions.

The exchange splitting M was set to 10 meV in both cases to account for the magnetisation. The Slater integrals F_{dd}, F_{pd}, and G_{dd} were set by default to 100% of the atomic values, i.e. 80% of the Hartree-Fock values. Also the spin-orbit couplings in both initial and final states were set to 100% of the value for Pd. For all simulations we used the same Lorentzian life time broadening, i.e. 0.3 eV at the M_3 edge and 1.2 eV at the M_2 edge. The instrumental (Gaussian) line broadening is 0.1 eV. To keep the number of fitting parameters reliably small, no charge transfer was included. Note, that the fitting parameters are not unique, i.e. there exist other sets of parameters yielding the same good agreement. For instance, the diamagnetic state is reached for reduced Slater integrals already with smaller crystal field splittings. This is particularly true for reduced values of F_{dd}, which represents a smaller Coulomb repulsion between d electrons and makes it less favourable to occupy higher energy levels. However, the spectral shape of the experimental diamagnetic XMCD can only be reproduced with crystal field splittings 10Dq in the range between +1.4 and +1.8 and F_{dd} between 0.8 and 1.1. For the paramagnetic XMCD the range of possible parameters is larger. However, it is reasonable to use the same value of 10Dq and slightly modify only the values of Dt and Ds to take into account the four additional oxygen anions in the close vicinity. The different contributions to the final fit are presented in **Supplementary Figure 3**. To show the influence of coordination symmetry leading to the paramagnetic state, in **Supplementary Figure 4** we present again the best fit assuming the "4+4" coordination explained above. For a better agreement with experimental data, the life time broadening has been slightly adjusted. In addition, simulations are presented with octahedral (six-fold) symmetry, i.e. for Ds = Dt = 0, for which a reasonable agreement between experiment and simulation can be found as well. This verifies, that the four additional oxygen anions can be described by two effective ligands, one above and one below the (x,y) plane. An eight-fold coordination like it was found for central 3d metal ions can be excluded, since the agreement between simulation and experiment is significantly worse as can be seen in Figure 4c. Particularly, the positive signal at the L_2 absorption edge cannot be reproduced.

In **Supplementary Table 2**, the sums of squared residuals for the three above mentioned symmetries are summarised according to:

$$\sigma^2 = \sum_{i=1}^{N} \left(x_{exp}(E_i) - x_{simu}(E_i)\right)^2 \quad (1)$$

where $x_{exp}(E_i)$ and $x_{simu}(E_i)$ denote the experimental and simulated value, respectively, at the same photon energy E_i. For the M_3 absorption edge, data between 527.5eV – 535.5eV were considered, for the M_2 absorption edge, data between 556.5 eV – 565 eV. In both cases, the number of points is 268. The squared residuals show that indeed the "4+4" model of a distorted square-planar symmetry fits best the experimental data.

Supplementary Note 3: X-ray linear dichroism

The x-ray (magnetic) linear dichroism – X(M)LD – has been calculated from simulated spectra as well. The Lorentzian line broadening was set to 0.3 eV for all three cases. To distinguish between the linear dichroism caused by different shapes of the corresponding unoccupied orbitals and its magnetic component, the exchange field representing an external magnetic field, was reduced from 10 meV to 1 meV. The resulting XANES, XMCD, and X(M)LD spectra at the Pd M_3 edge are shown in **Supplementary Figure 5**. For the diamagnetic state with the larger crystal field splitting (a), no field dependence is observed for the X(M)LD. Thus, it is a natural XLD reflecting the asymmetry of unoccupied orbitals. The XLD spectrum consists of one peak which is consistent with the simple picture of an unoccupied b_1 (d_{z2}) orbital, while all other orbitals are occupied and do not contribute to the absorption signal.

In the second diamagnetic state (Supplementary Figure 5b), the linear dichroism is reduced in amplitude and shows two peaks with opposite signs. The change of sign indicates that there exist two different unoccupied orbitals with different symmetry as it is the case for the b_1 (d_{z2}) and a_1 (d_{x2-y2}) orbital in the one-electron picture. This interpretation is in agreement with the observation that the paramagnetic state shows basically the same linear dichroism (Supplementary Figure 5c). Interestingly, reducing the exchange field from 10 meV to 1 meV changes the paramagnetic state to the intermediate diamagnetic state as can be clearly seen by the XMCD data.

As can be seen in Figure 1 of the main manuscript, the square-planar coordinated planes in a single polyoxopalladate cluster are aligned in different directions. Therefore, the x-ray linear dichroism will (partially) cancel out and the magnetic linear dichroism is too small to be measured.

Supplementary Note 4: Sum rules

As mentioned in the main manuscript, a quantitative determination of magnetic moments is not possible in a straight-forward manner due to the oxygen contribution to the XANES spectra.

Further limitations are (i) more delocalized electronic states, (ii) a possible contribution of the spin-dipole term, (iii) final states and multiplet effects.

The validity of XMCD sum rules for more itinerant systems was questioned for Pd-containing 3d transition metal alloys [29]. But due to the predominantly ionic character of the systems studied in the present work, this influence should be rather small.

The effective spin magnetic moment deduced from XMCD spectra contains the spin magnetic moment and the dipole moment of the spin density distribution, frequently called $\langle T_z \rangle$ term. The latter is known to be negligible for cubic symmetries only and can be sizeable in nanoscale materials. In the system studied here, it is partially cancelled due to the random orientation of the palladate cluster but does not vanish due to the large spin-orbit coupling [30]. In particular for the common diamagnetic state, a sizeable contribution may be present.

In addition, influences of final states and multiplet effects on the magnetic moments derived from XMCD were found for metal ions and may lead to an underestimation of spin magnetic moments up to about 20% depending on the absorbing ion, its electronic properties and crystal fields [31].

In **Supplementary Figure 6** the integrals of simulated 4d XANES and XMCD spectra (multiplied by 100) fitted to experimental data are shown for the sum-rule based analysis. The parameters *p*, *q*, and *r* denote the integral of XMCD over the M_3 absorption edge and over both, M_3 and M_2, absorption edges, respectively, following the notation of Chen et al. [12].

In **Supplementary Figure 7** the experimental XMCD and their integrals (again multiplied by 100) necessary for the sum-rule based extraction of magnetic moments are shown for samples (3) and (4). For the case of sample (3), $FePd_{12}(PhAs)_8$, the oxygen contribution represented by a Lorentzian line has been subtracted to analyse the Pd magnetism solely.

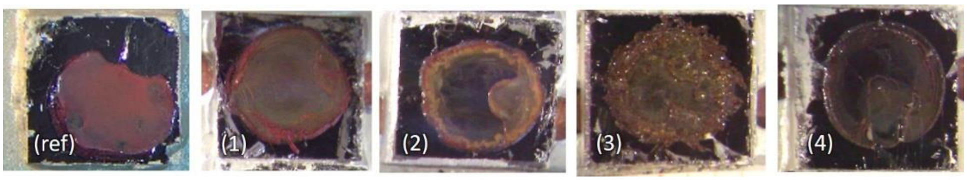

Supplementary Figure 1: Photographs of samples. Photographs of reference sample (ref) and palladate samples $CoPd_{12}P_8$ (1), $FePd_{12}P_8$ (2), $FePd_{12}(PhAs)_8$ (3), and $PdPd_{12}As_8$ (4) prepared on freshly cleaved HOPG and contacted with silver paste. The edge lengths of HOPG substrates are 5-6 mm. Samples were measured in homogeneously thin covered areas close to the centre.

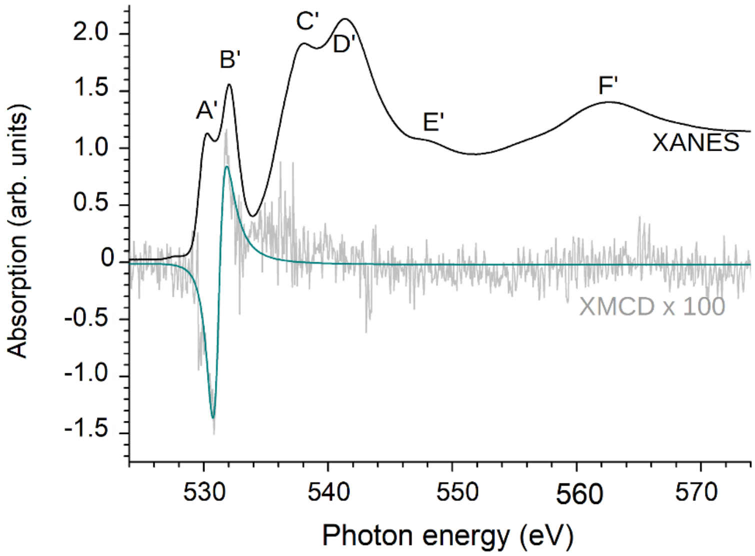

Supplementary Figure 2: Reference spectra at oxygen K edge. Oxygen K edge spectra of $CoFe_2O_4$ (without Pd contributions) used as qualitative reference for XANES (black line) and XMCD (grey line) of oxygen in a static magnetic field of 6T at a temperature of 7 K. The XMCD spectrum is multiplied by a factor of 100. The blue line corresponds to peak fitting using two Lorentzian lines.

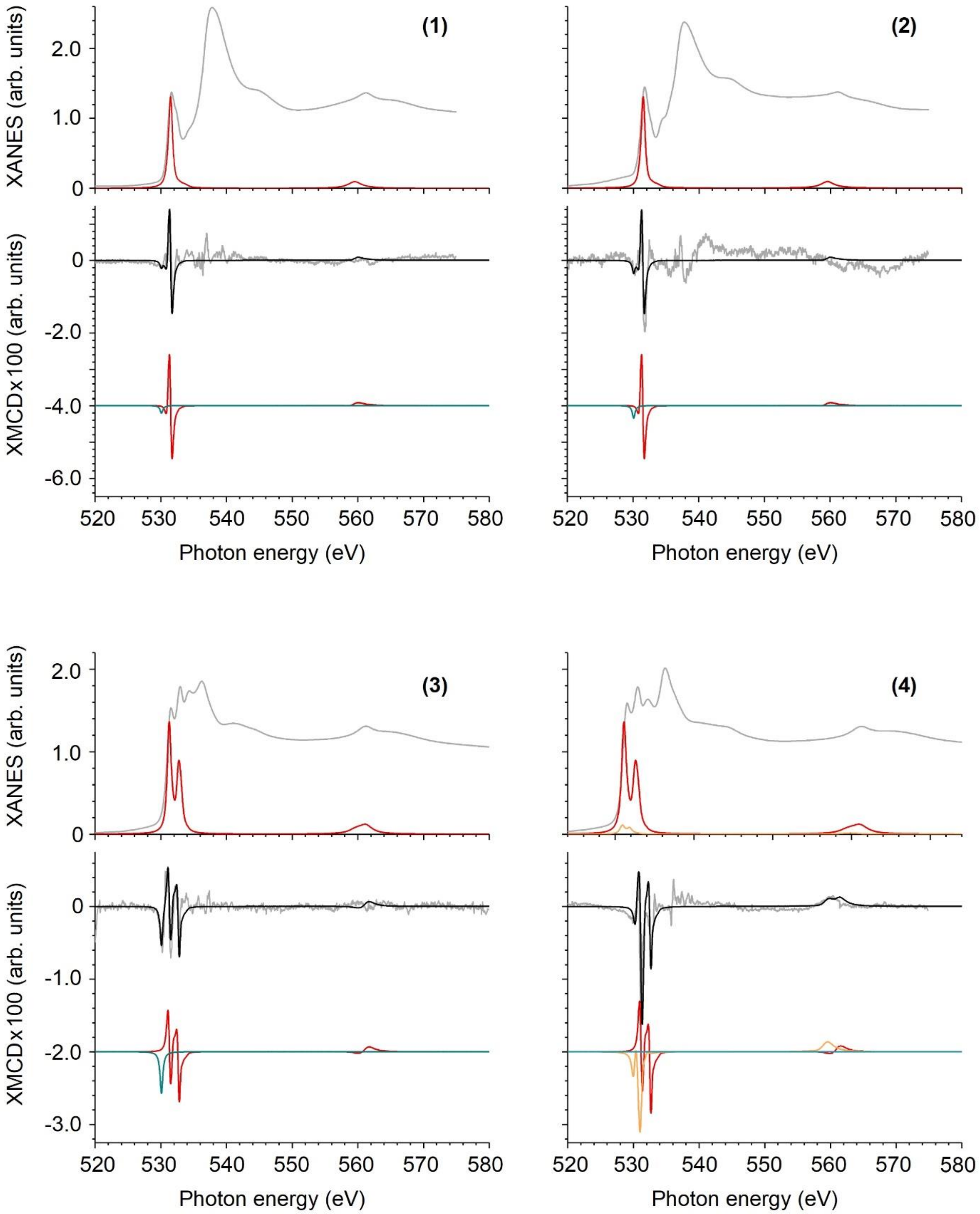

Supplementary Figure 3: Discriminating different contributions to absorption spectra by simulations. Simulations of 4d XANES and XMCD in comparison to the experimental data (grey lines, cf. Fig. 2) for $CoPd_{12}P_8$ (1), $FePd_{12}P_8$ (2), $FePd_{12}(PhAs)_8$ (3) and $PdPd_{12}$ (4). Red lines refer to the contribution of Pd ions in the polyoxopalladate shell, orange lines to the contributions of central Pd ions, and blue lines to oxygen contributions to the simulated spectra. Black lines correspond to the sum of all contributions to the total XMCD. Vertical shifts added for better visibility. Note the different ordinate scale of (1), (2) with respect to (3), (4).

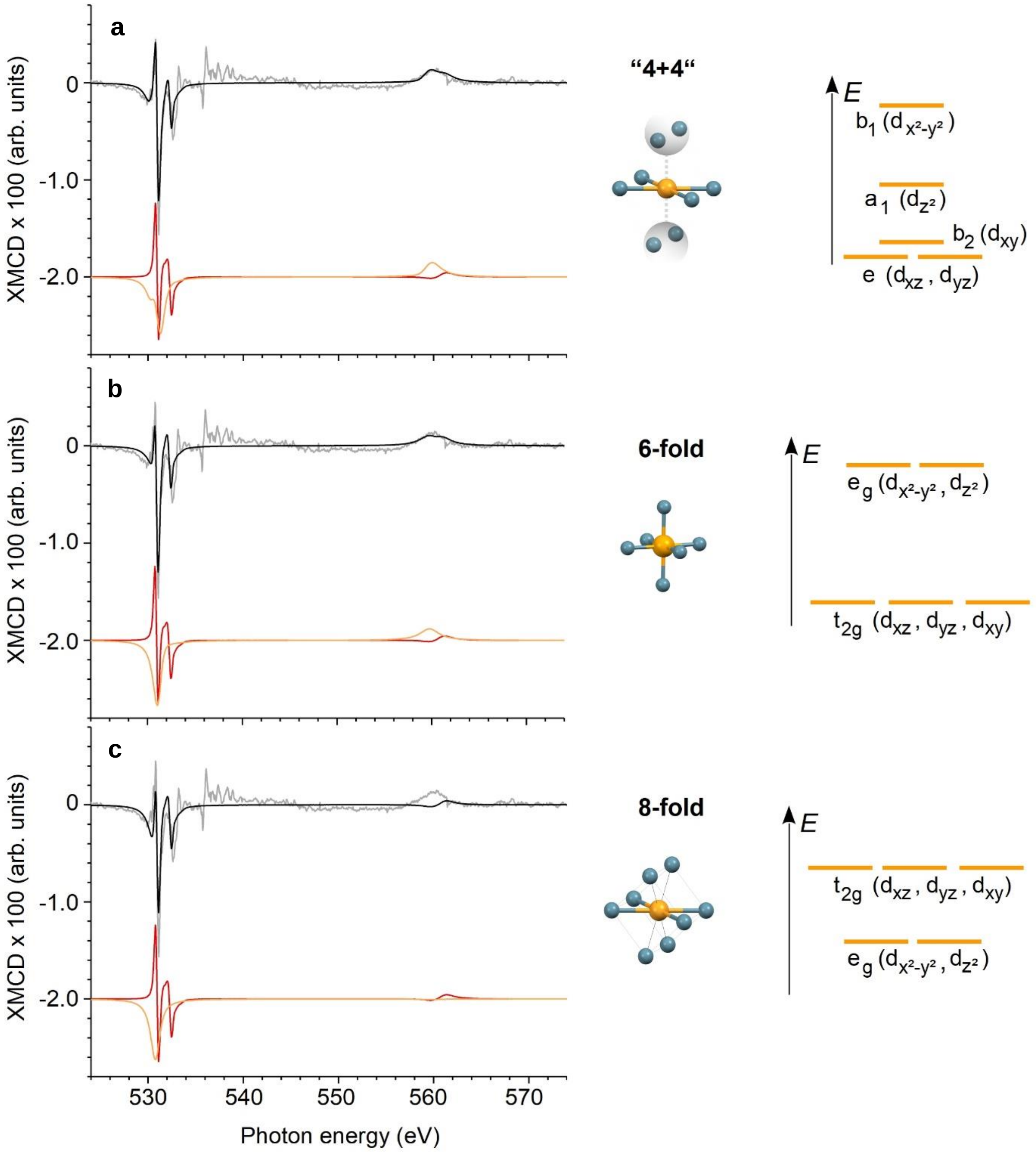

Supplementary Figure 4: Simulations with different coordination symmetries. Simulations of palladium(II) XMCD signals (black lines) as a sum of shell ions (red lines) and central ions (orange lines) compared to experimental data (grey lines). The central ions were placed in a tetragonal distorted environment “4+4” (a), six-fold octahedral coordination (b) or tetrahedral coordination representing an 8-fold symmetry (c). Corresponding splitting of energy levels are sketched in addition. Note that these diagrams are limited to one-electron states but used here for illustration.

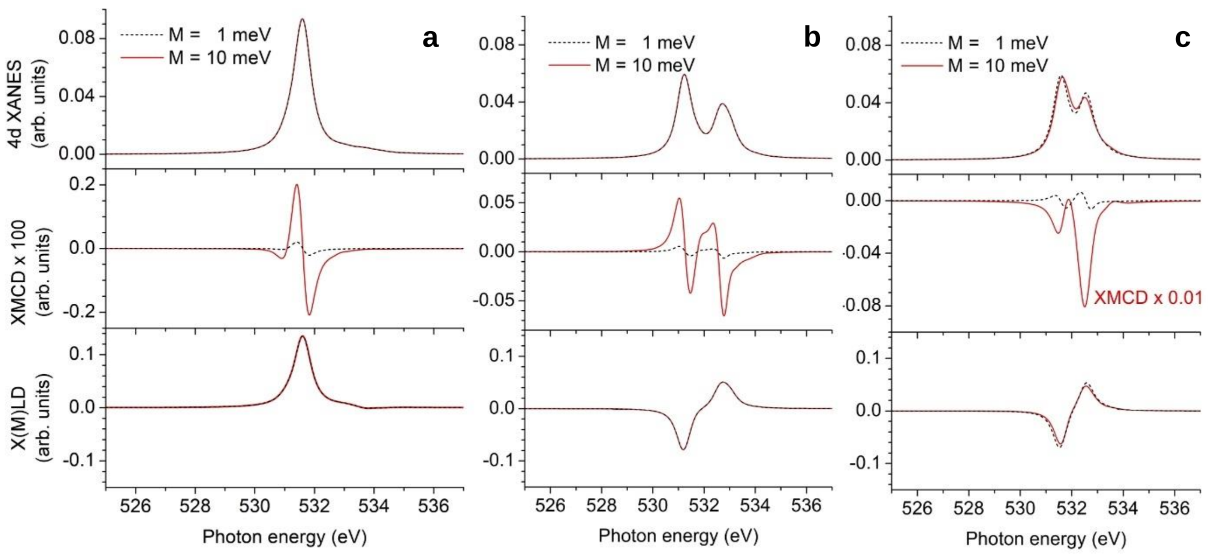

Supplementary Figure 5: Simulations of 4d XANES, dichroic signals and their field dependences. Field-dependent simulations of Pd M_3 XANES, XMCD, and X(M)LD for the three different configurations used in this work to fit experimental spectra. For easier comparison, the Gaussian line broadening is 0.3 eV, the Lorentzian line broadening is 0.1 eV in all cases.

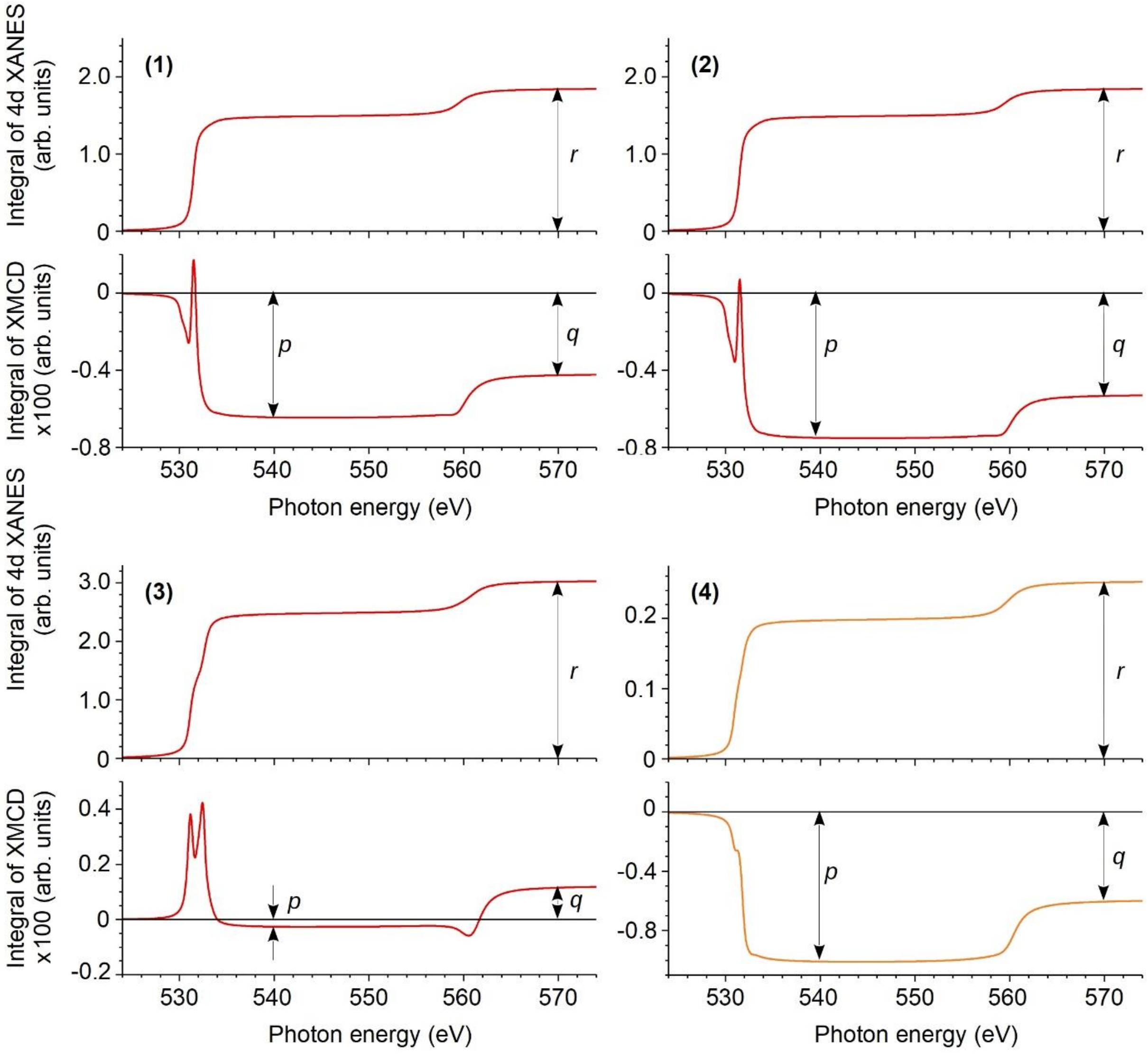

Supplementary Figure 6: Integration for sum rule-based estimation of magnetic moments from simulated spectra. Integrals of simulated Pd-4d XANES and XMCD spectra after fitting to experimental data for palladium(II) in the shell of $CoPd_{12}P_8$ (1), $FePd_{12}P_8$ (2), $FePd_{12}(PhAs)_8$ (3) and in the centre of $PdPd_{12}$ (4).

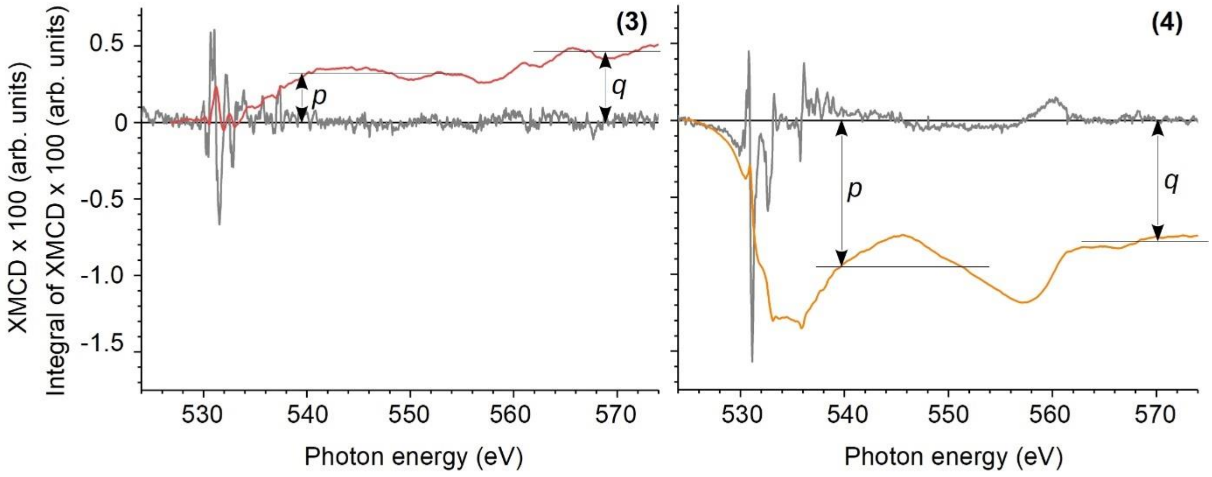

Supplementary Figure 7: Integration for sum rule-based estimation of magnetic moments from experimental spectra. XMCD spectra (grey) and integrals of XMCD of Pd ions in (3) $FePd_{12}(PhAs)_8$ (red line) and (4) $PdPd_{12}As_8$ (orange line).

Parameter	$CoPd_{12}P_8$, $FePd_{12}P_8$ Pd^{2+} in shell	$FePd_{12}(PhAs)_8$, $PdPd_{12}As_8$ Pd^{2+} in shell	$PdPd_{12}As_8$ central Pd^{2+}
10Dq (eV)	1.9	1.7	1.7
Dt (eV)	0.109	0.097	0.07
Ds (eV)	0.287	0.256	0.15
M (meV)	10	10	10
F_{dd}	0.8	1.0	1.0
F_{pd}	1.0	1.0	1.0
G_{dd}	1.0	1.0	1.0
SO	1.0	1.0	1.0
$\Delta E(M_3)$ (eV)	0.3	0.3	0.3
$\Delta E(M_2)$ (eV)	1.2	1.2	1.2
ΔE (eV)	0.1	0.1	0.1

Supplementary Table 1: Fitting parameters. Summary of parameters for simulations using the CTM4XAS program package as presented in Figures 5 and 6. 10Dq, Ds, and Dt describe the crystal field, M the exchange field, F_{dd}, F_{pd}, and G_{pd} the Slater integrals with respect to the atomic values, SO the relative spin-orbit couplings (core and valence), $\Delta E(M_3)$ and $\Delta E(M_2)$ the Lorentzian life-time broadenings and ΔE the Gaussian instrumental broadening. Charge transfer has not been included.

Model	$\sigma^2(M_3)$ (arb. units)	$\sigma^2(M_2)$ (arb. units)
Distorted square-planar, “4+4“	3.01	0.165
Octahedral, 6-fold	3.95	0.165
Tetrahedral, 8-fold	7.19	1.23

Supplementary Table 2: Squared residuals. Sums of squared residuals in the range of M_3 or M_2 absorption edge between the experimental XMCD data (multiplied by 100) and CTM4XAS simulations using different coordination symmetries. Spectra are presented in Supplementary Figure S3. The number of points is 268 for both $\sigma^2(M_3)$ and $\sigma^2(M_2)$.

Supplementary References

[26] Suntivich J, Hong W T, Lee, Y-L, Rondinelli J M, Yang W, Goodenough J B, Dabrowski B, Freeland J W, Shao-Horn Y. Estimating Hybridization of Transition Metal and Oxygen States in Perovskites from O K-edge X-ray Absorption Spectroscopy. J. Phys. Chem. 2014;118:1856-63.

[27] Sinha A K, Singh M N, Achary S N, Sagdeo A, Shukla D K, Phase D M. Crystal field splitting and spin states of Co ions in cobalt ferrite with composition $Co_{1.5}Fe_{1.5}O_4$ using magnetization and X-ray absorption spectroscopy measurements. J. Magnet. Magnet. Mater. 2017;435:87-95.

[28] Wu Z Y, Gota S, Jollet F, Pollak M, Goutier-Soyer M, Natoli C R. Characterization of iron oxides by x-ray absorption at oxygen K-edge using a full multiple scattering approach.Phys. Rev. B 1997;55:2570-7.

[29] Miyahara T, Park S-Y, Hanyu T, Hatano T, Moto S, Kagoshima Y. Comparison between 3p and 2p magnetic circular dichroism in transition metals and alloys: Is the sum rule applicable to itinerant magnetic systems? Rev. Sci. Instrum. 1995;66 (2):1558-60.

[30] Ederer, C, Komelj, M,Davenport, J W, Fähnle M. Comment on the analysis of angle-dependent X-ray magnetic circular dichroism in systems with reduced dimensionality. J. Electron Spectrosc. Relat. Phenom. 2003;130:97-100.

[31] Piamonteze C, Miedema P, de Groot F M F. Accuracy of the spin sum rule in XMCD for the transition-metal L edges from manganese to copper. Phys. Rev. B 2009;80:184410.
