# Supplementary material for: Intramolecular crossover from unconventional diamagnetism to paramagnetism of palladium ions probed by soft X-ray magnetic circular dichroism
Source: Commun Chem. 2020 Jul 31;3:96. doi: 10.1038/s42004-020-0327-9 (PMC9814631; doi:10.1038/s42004-020-0327-9)
Supplement: Supplementary file 2 — Description of Additional Supplementary Files [file 42004_2020_327_MOESM2_ESM.pdf]

Description of Additional Supplementary Files

File Name: Supplementary Movie 1
Description: Spin density of $CoPd_{12}P_8$. Animated rotation of the calculated spin density isosurface (0.01 r_B^{-1}) of $CoPd_{12}P_8$ (yellow). For clarity, the crystal structure is sketched in addition (Pd in red, O in blue, P in grey).

File Name: Supplementary Movie 2
Description: Spin density of $PdPd_{12}As_8$. Animated rotation of the calculated spin density isosurface (0.01 r_B^{-1}) of $PdPd_{12}As_8$ (yellow). For clarity, the crystal structure is sketched in addition (Pd in red, O in blue, As in grey).
